# Myopathic Carnitine Palmitoyltransferase II (CPT II) Deficiency: A Rare Cause of Acute Kidney Injury and Cardiomyopathy

**DOI:** 10.7759/cureus.46595

**Published:** 2023-10-06

**Authors:** Efrain Castillo, Debbie Medina, Nick Schoenmann

**Affiliations:** 1 Medicine, Universidad Latina de Panamá, Panama City, PAN; 2 General Medicine, Universidad Latina de Panamá, Panama City, PAN; 3 Emergency Medicine, Augusta University Medical College of Georgia, Augusta, USA

**Keywords:** carnitine o-palmitoyltransferase, inborn errors of metabolism, fatty acid, renal replacement therapy, myoglobinuria, acute kidney injury, rhabdomyolysis

## Abstract

Carnitine palmitoyltransferase II (CPT II) deficiency is a long-chain fatty acid (LCFA) oxidation disorder. There are three main types classified by symptoms and age of onset: the neonatal form, the infantile hepatocardiomuscular form, and the adult or myopathic form. The first two are early-onset severe disorders presenting with marked hypoketotic hypoglycemia, cardiomyopathy, and liver dysfunction. The latter is characterized by muscle pain and weakness and stiffness, typically triggered by exercise or febrile illnesses and occasionally associated with myoglobinuria. One of the most common complications is acute kidney injury (AKI) following massive rhabdomyolysis, which is managed with aggressive fluid therapy; crystalloid solutions are preferred.

We report an otherwise healthy 38-year-old patient who presented with severe myalgia, cramps, fatigue, low-grade fever, and transient myoglobinuria, after intense physical training. Significant recurrent muscle pain was reported. Family history was unremarkable. Imaging studies showed no abnormalities. Echocardiogram showed a left ventricle ejection fraction (LVEF) of 40%. Acetylcarnitine analysis with tandem mass spectrometry and molecular tests confirmed the diagnosis. Fluid resuscitation was started. Acute kidney injury was diagnosed and managed with plasmapheresis and five sessions of hemodialysis. The patient was discharged upon the improvement of renal function with lifestyle modification recommendations. In otherwise healthy young adults presenting with myalgia and rhabdomyolysis triggered by physical activity or infection, CPT II deficiency should be considered, and genetic testing should be initiated to provide an opportunity for patients to modify their daily lifestyle, preventing future attacks and the development of complications.

## Introduction

Carnitine palmitoyltransferase II (CPT II) deficiency is a relatively rare genetic disorder with approximately more than 300 cases reported [1]. CPT II deficiency has an autosomal recessive inheritance pattern that affects the cellular metabolism of fatty acids [2]. Long-chain fatty acids (LCFA) do not diffuse passively to the mitochondria; their transportation from the cytoplasm requires the carnitine enzyme complex. There are three main genetic disorders involving the cellular transportation of LCFA: carnitine palmitoyltransferase I (CPT I) deficiency, carnitine-acylcarnitine translocase (CACT) deficiency, and CPT II deficiency [3].

Physiologically, CPT II regulates the transformation of long-chain acylcarnitine to acyl-coenzyme A (CoA) in the inner mitochondrial membrane for β-oxidation [4], which is a catabolic process in which fatty acid chains are cleaved in the mitochondria yielding acyl-CoA and acetyl-CoA leading to adenosine triphosphate (ATP) production through the citric acid cycle, becoming vital not only for the use of stored fats as an energy source but also for offering an alternative fuel source for various tissues during fasting and during periods of high energy demand or low glucose availability through ketone body production.

LCFA are the key source of energy for the cardiac, hepatic, and musculoskeletal tissues, especially during episodes of metabolic stress. In CPT II deficiency, any physiological stressful state, such as fasting, exposure to cold temperatures, stress, infections, and prolonged exercise, can lead to myocyte energy depletion and subsequent rhabdomyolysis. There are three phenotypes of CPT II deficiency: the myopathic form, the infantile hepatocardiomuscular form, and the fatal neonatal form [5]. We present a patient with a late diagnosis of myopathic CPT II deficiency and a diminished quality of life due to recurrent myalgias that later progressed to acute kidney injury (AKI) and cardiomyopathy that were discovered in the emergency department (ED), making this case unique since there are no reports of these two complications occurring simultaneously in a patient with years of symptomatic undiagnosed disease.

## Case presentation

We present a 38-year-old female who was transferred to the emergency department (ED) from a primary care clinic, due to recurrent episodes of rhabdomyolysis suspected to be the result of an underlying hereditary metabolic myopathy. Upon arrival at the ED, the patient presented with mild dyspnea, severe muscle pain, fatigue, low-grade fever, transient myoglobinuria, and continuous and involuntary cramping localized to the lower limbs. Past medical history revealed that three days prior to the onset of symptoms, she had engaged in vigorous physical activity for three months in preparation for a marathon with an approximate completion of 40 miles weekly and three days of rest a week. No family history was disclosed. She was treated with IV analgesics, reported symptomatic improvement, and was sent home with oral nonsteroidal anti-inflammatory drugs (NSAIDs) for five days. The next day, she returned to the ED with worsening dyspnea at rest and a fever of 40.2°C, managed with parenteral NSAIDs. She was later admitted to the nephrology department with the diagnosis of acute kidney injury. A review of systems revealed nasal congestion, malaise, weakness, and muscle pain with a predominance of the distal muscles group. Immunological markers including antinuclear antibodies (ANA), anti-DNA, cytoplasmic antineutrophil cytoplasm antibodies (cANCA), and blood cultures yielded negative results. Antibodies against atypical pathogens and respiratory viruses (Pneumoslide) were positive for immunoglobulin M (IgM) *Rhinovirus*. There were increased levels of fibrinogen and C-reactive protein associated with leukocytosis, creatine kinase (CK) levels of 90,000 IU, creatine kinase myocardial band (CK-MB) of 50 IU, peak myoglobin of 250,00 IU, and lactate dehydrogenase (LDH) of 2,300 U/L. The two-step serologic approach for Lyme was negative. Urinalysis showed high levels of myoglobin; urine culture was sterile.

The patient’s initial serum creatinine was 5.4 mg/dL (Table 1) for which she was initially treated with IV fluids and later required plasmapheresis and hemodialysis due to refractory acute kidney injury (AKI), cardiomyopathy volume overload, and impending respiratory insufficiency. The first hemodialysis session was performed through a temporary femoral venous catheter in the emergency department-intensive care unit (ED-ICU). At 36 hours, the patient required non-invasive ventilatory support due to worsening hypoxemic respiratory failure, suspected to be the result of systemic inflammation and prolonged increased work of breathing.

**Table 1:** Main laboratory data on admission. WBC, white blood cell; TSH, thyroid-stimulating hormone; T3, triiodothyronine; T4, thyroxine; ESR, erythrocyte sedimentation rate; CRP, C-reactive protein; CK, creatinine kinase; CK-MB, creatine kinase myocardial band; BUN, blood urea nitrogen; SCr, serum creatinine; Na, sodium; K, potassium; Ca, calcium; ALT, alanine aminotransferase; AST, aspartate aminotransferase

Laboratory tests	Value	Reference range
WBC count, ×10^3^/μL	13.5	4.5-11.0
Segmented neutrophils, %	60	54-62
Bands, %	2	3-5
Eosinophils, %	1.7	1-3
Basophils, %	0.34	0-0.75
Monocytes, %	6.1	3-7
Lymphocytes, %	29.8	25-33
TSH, μU/mL	3.3	0.4-4.0
T3, ng/dL	128	100-200
T4, µg/dL	5.3	5-12
Glucose, mg/dL	59	Fasting: 70-100
ESR, mm/hour	27	Women: 0-20; male: 0-15
CRP, mg/dL	1.5	<1.0
CK, U/L	90,434	Male: 25-90; female: 10-70
CK-MB, U/L	50	5-25
BUN, mg/dL	26	7-18
SCr, mg/dL	5.4	0.6-1.2
ALT, U/L	54	10-40
AST, U/L	49	12-38
Na, mEq/L	139	135-145
K, mEq/L	5.6	3.5-5.0
Ca, mEq/L	8.1	8.4-10.2

**Figure 1:**
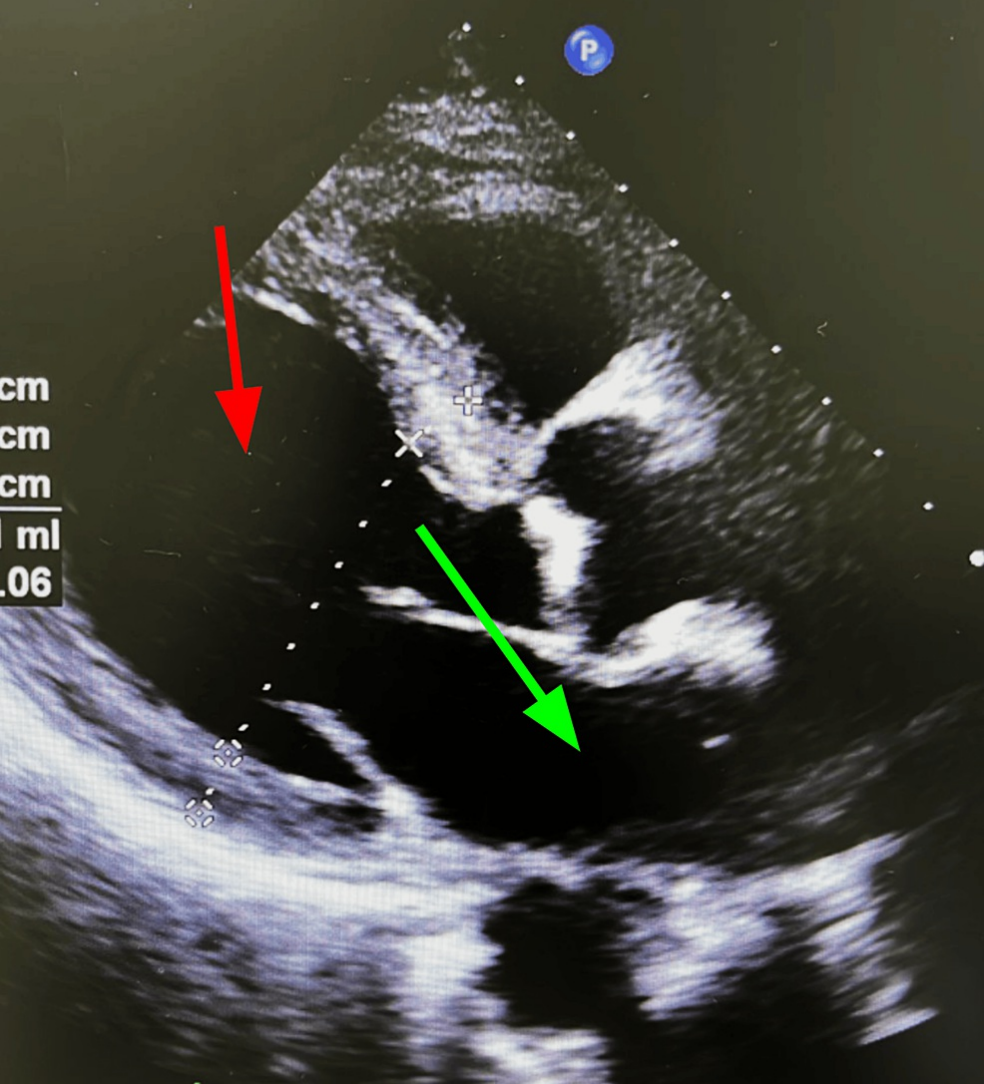
Echocardiogram with suggestive features of dilated cardiomyopathy. Left ventricular diastolic diameter of 48 mm, left ventricular systolic diameter of 40 mm, and LVEF of 45% (red arrow) and left atrial enlargement (green arrow). LVEF: left ventricle ejection fraction

Capillary blood glucose monitoring revealed only mild hypoglycemia. Renal ultrasound showed normal-sized kidneys with no abnormalities. Chest X-ray did not show pulmonary congestion, cardiomegaly, or other pathological findings. A point-of-care ultrasound (POCUS) was performed at the ED, which showed a slight enlargement of the left ventricle (LV) without organomegaly or other pathological data. An echocardiogram was requested, revealing left atrial enlargement and left ventricular eccentric hypertrophy with an LV ejection fraction of 45% (Figure 1), for which a cardiology consult was done.

**Figure 2:**
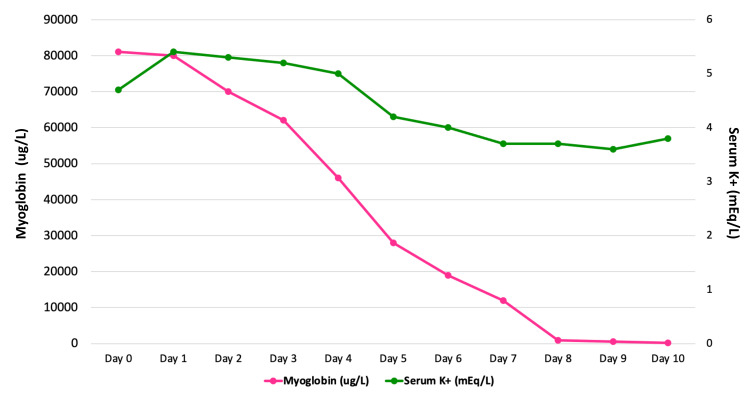
The evolution of myoglobin and serum potassium (K) values ​​during hospitalization.

On admission, CK peaked at 90,000 U/L, which ​​progressively declined; CK levels were at 200 U/L upon discharge (see Table 2). Serum potassium peaked at 5.4 mEq/L on day 1 with a gradual decrease (Figure 2). Following admission, a 10% glucose infusion was started at 2 mL/kg/hour. Fluid therapy with lactated Ringer’s (LR) was initiated 24 hours later at 80 mL/hour. The patient did not present with electrolyte disturbances prior to or after initiating fluid therapy.

**Table 2:** Laboratory findings during the hospitalization of our patient. K, potassium; LDH, lactate dehydrogenase; Mb, myoglobin; CK, creatinine kinase; AST, alanine aminotransferase; AST, aspartate aminotransferase; SCr, serum creatinine

Laboratory test	Day 0	Day 1	Day 2	Day 3	Day 4	Day 5	Day 6	Day 7	Day 8	Day 9
K, mEq/L	4.7	5.4	5.3	5.2	5.0	4.2	4.0	3.7	3.7	
LDH, U/L			2,340	1,432		431	460		331	
Mb, ug/L		>10,000		>5,000				>1,200	134.6	
CK, U/L	45,000	90,000	72,000	56,000	37,000	21,000	16,000	3,100	750	200
AST, U/L		2,600	2,000	1,404		460	300	115	96	
ALT, (U/L)		1,100	910	546		307	115	74		
SCr, mg/dL	5.4	6.1	5.84	6.22	5.6	7.0	7.4	6.0	5.6	4.2
Urine output (mL/24 hours)	200	200	350	410	500	1,700	1,900	2,700	3,000	4,500

**Figure 3:**
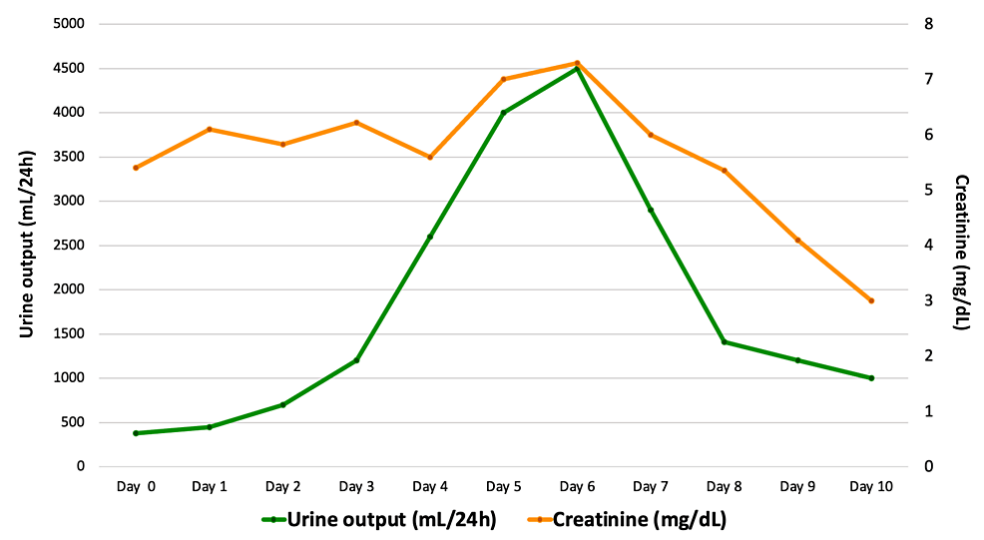
Serum creatinine levels and diuresis during the hospitalization period.

During the hospitalization period, five hemodialysis sessions were completed. Renal replacement therapy was discontinued on day 8 following a favorable clinical progression and gradual reduction in serum creatinine and blood urea nitrogen and the normalization of the urine output after experiencing the anuric and polyuric phases of AKI (see Figure 3). Our patient received 2 mL/kg/hour of 10% dextrose (200 mg/kg), keeping in mind the end goal of avoiding the characteristic hypoglycemic episodes of fatty acid disorders. A total of 16.2 L of lactated Ringer’s was given upon the completion of therapy, when there was a sufficient reduction in CK values ​​and a clear improvement clinically and in renal function.

Acylcarnitine analysis was performed using tandem mass spectrometry, revealing peaks of C12:0 (lauroylcarnitine), C16:0 (palmitoylcarnitine), and C18:1 (oleoylcarnitine). These tests are crucial because they provide concrete evidence based on clinical suspicion, for detecting other metabolic abnormalities, monitoring the disease, and guiding treatment. Analyses are reliable because acylcarnitines are intermediates in the transport of fatty acids for β-oxidation. Our patient’s findings were suggestive of a CPT II deficiency. Molecular genetic testing yielded a mutation with the change of the DNA nucleotide c.1342T>C (exon 4) and modification in the p.Phe448Leu protein, which is classified as a pathogenic variant registered in the Leiden Open Variation Database. The identification of these specific mutations is key for confirming the diagnosis and understanding the genetic basis for genetic counseling and family planning.

Upon discharge, a diet low in long-chain triglycerides and high in carbohydrates and medium-chain fatty acids was recommended. The patient was advised to increase the carbohydrate and fluid intake in cases of increased metabolic demand (febrile illness and exercise) to prevent flares. Lifestyle modifications such as avoiding general anesthesia, drugs such as ibuprofen and valproate, and strenuous physical activities, as well as prolonged fasting avoidance, were encouraged. Daily bezafibrate in doses of 400 mg was given for three months. The renal and hepatic functions were monitored during this period with no abnormalities. There was a remarkable symptomatic improvement reported in the first, third, and sixth months after discharge. Genetic counseling was also provided to the patient and her partner prior to conception.

## Discussion

**Figure 4:**
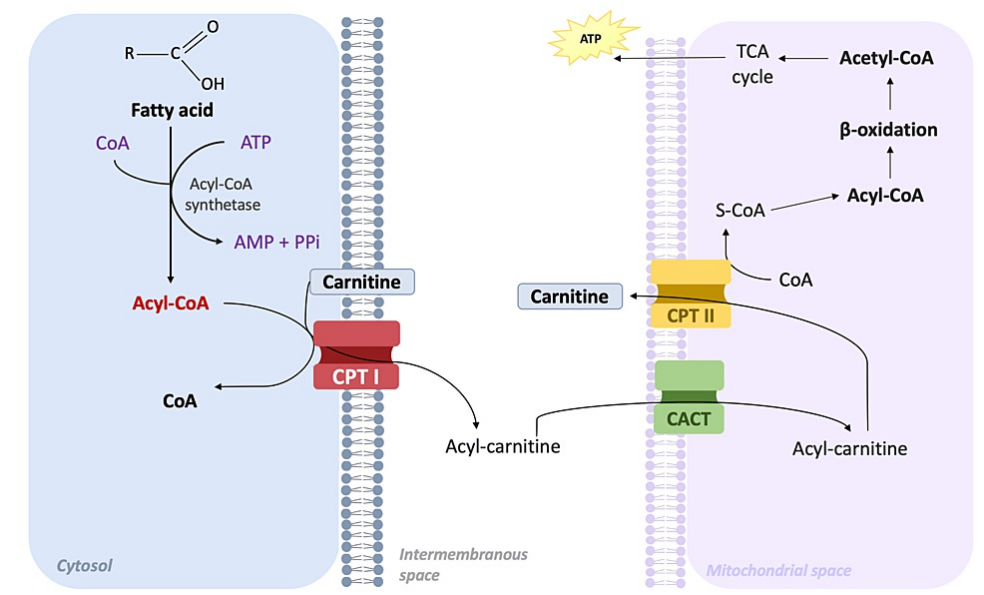
Cellular pathway showing the carnitine and acylcarnitine transportation from the cytosol to the mitochondrial matrix. The image was created by Castillo and reviewed by Schoenmann, the authors of this article. CoA, coenzyme A; ATP, adenosine triphosphate; AMP, adenosine monophosphate; PPi, pyrophosphate; CPT, carnitine palmitoyltransferase; TCA, tricarboxylic acid; CACT, carnitine-acylcarnitine translocase

CPT I in the outer mitochondrial membrane catalyzes the first step by converting long-chain acyl-CoA with carnitine to long-chain acylcarnitine and coenzyme A [6]. The acylcarnitine available in the intermembranous space is transferred to the inner mitochondrial membrane via CACT, to undergo the reverse reaction where CPT II catalyzes the dissociation of acylcarnitine into acyl-CoA and carnitine. After this reaction, the acyl-CoA produced is available for β-oxidation [7]. The released carnitine is available for the transportation of fatty acids (Figure 4). In cases of CPT II deficiency, the cytotoxic effects are produced by an increase in cytoplasmic and mitochondrial ionized calcium as a result of ATP depletion and/or direct damage to the plasma membrane [8].

Our patient is a confirmed case of CPT II deficiency, a rare autosomal recessive disorder that affects long-chain fatty acid oxidation. Three phenotypes of CPT II deficiency are described in the literature: the neonatal form, the severe infantile form, and the more common but less severe myopathic form [9,10]. Our patient met the clinical diagnostic criteria for the myopathic form of CPT II deficiency. In our case, these symptoms included a late presentation with exercise-induced muscle pain, weakness, and associated myoglobinuria [11]. The symptoms appeared after a fever due to an upper respiratory infection and intense physical activity.

The myopathic form of CPT II deficiency, although a rare condition, is the most common disorder of lipid metabolism affecting musculoskeletal tissue and the most common cause of hereditary myoglobinuria. More than 75% of the reported cases are males. It can manifest anywhere from infancy to early adulthood. The clinical presentation is characterized by multiple transient episodes of severe or exercise-induced myalgia, which may or may not be accompanied by myoglobinuria. Myoglobinuria with red or brown urine occurs during attacks in 75% of cases, commonly precipitated by prolonged exercise, febrile illness, and fasting [12]. Physical examination usually does not reveal signs of myopathy (muscle weakness, severe pain, and elevated creatinine kinase) during and between attacks, with only less than 10% of cases presenting with persistent myopathy [13]. During attacks, the duration and intensity of pain are highly variable, ranging from minimal proximal muscle discomfort to complications with acute kidney injury from rhabdomyolysis [14].

In this case, our patient required hemodialysis therapy for acute kidney injury caused by massive rhabdomyolysis, mild acute liver injury, and respiratory failure that was resolved with non-invasive ventilation. The diagnosis of rhabdomyolysis is not always obvious because less than 15% of cases present with the classic triad of myalgias, generalized weakness, and darkened urine. In such cases, it is necessary to determine the cause and begin early management. For the diagnosis of CPT II deficiency, the medical team must have a high suspicion for such an unusual cause of persistent myalgias.

Plasma acylcarnitine profile analysis has a higher sensitivity for diagnosing disorders associated with an accumulation of long-chain acylcarnitines, such as the case of our patient, than other diagnostic measures such as amniotic fluid, blood dried spot, cell culture medium, or urine samples [15]. The objectives of the treatment of CPT II deficiency are the prevention of renal failure, the decrease in the frequency of episodes, and the correction of acid-base disorders since there is currently no definitive treatment. The treatment of CPT-II deficiency entails strict dietary modifications; a diet low in fat (20%) but covering essential fatty acids and rich in long-chain carbohydrates is generally recommended. Patients should follow a diet composed of 70% carbohydrates to prevent episodes of hypoglycemia and arrhythmias associated with fatty acid disorders [16]. It is recommended to avoid precipitants such as intense physical activity, certain medications (ibuprofen, diazepam, and valproic acid), general anesthesia, and stress [17]. Some studies have found benefits in the use of l-carnitine as part of the treatment, but this remains controversial and is likely outside the scope of a primary care physician.

In cases of rhabdomyolysis, such as that of our patient, treatment is based on the aggressive administration of crystalloid fluids. It is acceptable to start fluid resuscitation in patients with rhabdomyolysis with either lactated Ringer’s solution or saline (0.9% or 0.45%). A starting infusion at a rate of 400 mL/hour is generally recommended. If signs of hypovolemia are evident, it is advised to increase fluids to 1000 mL/hour. Urine output should be monitored with a goal-directed therapy ranging from 1 to 3 mL/kg/hour and up to 300 mL/hour with hourly reassessment resolved [18].

During monitoring, patients may present with increased urine output (≥200 mL/hour), in which case urinary pH should be assessed. For a pH of ≥6.5, it is recommended to continue fluid resuscitation with constant evaluation of blood volume within the first hour; urinary pH with arterial blood gas at the fourth hour; CK at the sixth hour; and phosphate, magnesium, complete blood count, and serum and urinary osmolarity at the 24th hour. If the urinary pH is <6.5, hypertonic (8.4%) sodium bicarbonate is indicated at 2 mL/kg for one hour with serum sodium and calcium monitoring, before proceeding with fluid resuscitation. In cases where, after the initial resuscitation, patients present with a urine output lower than 200 mL/hour, it is strongly suggested to consider renal replacement therapy in four particular situations: oligo-anuria, refractory hyperkalemia, volume overload, and in cases of refractory metabolic acidosis.

## Conclusions

CPT II deficiency leads to limited removal of carnitine from LCFA causing absent or minimal fatty acid oxidation and producing no substrate for gluconeogenesis and ketogenesis that leads to the classic hypoketotic hypoglycemia. Because there is an accumulation of LCFA and acylcarnitines, deposition and damage to various tissues and complications such as congestive heart failure, acute kidney injury, hyperammonemia, and multiple organ failure can arise. All fatty acid oxidation disorders require a high degree of suspicion in the emergency setting. We aim to highlight the presentations of young adults with recurrent episodes of myalgias, sometimes associated with myoglobinuria, muscle stiffness, exercise intolerance, and cardiac abnormalities, that are refractory to standard therapy, even in an adult-onset CPT II deficiency.
